# Application of Deep Convolutional Neural Networks in the Diagnosis of Osteoporosis

**DOI:** 10.3390/s22218189

**Published:** 2022-10-26

**Authors:** Róża Dzierżak, Zbigniew Omiotek

**Affiliations:** Department of Electronics and Information Technology, Lublin University of Technology, ul. Nadbystrzycka 38A, 20-618 Lublin, Poland

**Keywords:** osteoporosis, convolutional neural networks, deep learning, VGG16, image classification, neural networks

## Abstract

The aim of this study was to assess the possibility of using deep convolutional neural networks (DCNNs) to develop an effective method for diagnosing osteoporosis based on CT images of the spine. The research material included the CT images of L1 spongy tissue belonging to 100 patients (50 healthy and 50 diagnosed with osteoporosis). Six pre-trained DCNN architectures with different topological depths (VGG16, VGG19, MobileNetV2, Xception, ResNet50, and InceptionResNetV2) were used in the study. The best results were obtained for the VGG16 model characterised by the lowest topological depth (ACC = 95%, TPR = 96%, and TNR = 94%). A specific challenge during the study was the relatively small (for deep learning) number of observations (400 images). This problem was solved using DCNN models pre-trained on a large dataset and a data augmentation technique. The obtained results allow us to conclude that the transfer learning technique yields satisfactory results during the construction of deep models for the diagnosis of osteoporosis based on small datasets of CT images of the spine.

## 1. Introduction

Due to the fact that we live in an aging society, osteoporosis has become a disease of serious concern around the world. According to the WHO definition, osteoporosis is a systemic skeletal disease characterised by low bone mass, impaired microarchitecture of bone tissue and, consequently, increased fragility and susceptibility to fractures. In 2019, the number of cases in Europe increased to 32 million (5.6% of the total European population aged 50+) [1]. The development of this disease is influenced by lifestyle, especially diet and physical activity. Thus, it can be expected that the pandemic will worsen the situation and significantly affect the progression of the disease in patients. According to reports [1], the incidence rate will increase significantly in the near future. It should be remembered that, in the last two years, patients were referred to osteoporosis-dedicated follow-up examinations much less frequently [2], and, therefore, so many patients are still unaware of their disease. Most often, osteoporosis is diagnosed at an advanced stage, when osteoporotic fractures occur. This is especially dangerous for the spine. Therefore, continuous research is necessary in order to develop the best diagnostic method that would allow the detection of osteoporosis at an early stage of development [3]. The general trend of research in this area shows that the best results are obtained from the analysis of bone tissue microarchitecture [2,4,5,6,7,8,9].

In recent years, a rapid development of machine learning algorithms has taken place, especially deep learning methods. Deep learning is a machine learning concept based on artificial neural networks [10]. In most cases, they use multiple layers of interconnected neural networks. There are many types of architecture of such networks, including deep belief networks, convolutional networks, recursive networks, long short-term memories, deep Boltzmann machines, and deep coding networks. Many of them work very well in the process of image recognition, including biomedical images. Recent studies report the use of artificial neural networks in the diagnostics of intramucosal gastric cancer [11], early detection and prediction of chronic kidney disease [12], major depressive and bipolar disorders [13], as well as COVID-19 pneumonia [14]. There are also publications on new methods of diagnosing osteoporosis with the use of deep learning [15,16,17].

One of the important issues in building machine learning models (including deep models), is the estimation of uncertainty as a measure of confidence in the model’s predictions. This problem, among others in the area of computer vision, is successfully addressed by using Bayesian convolutional neural networks (BCNNs). Networks of this type prevent over-fitting by introducing uncertainty estimation. BCNNs are preferred in problems where CNNs are an appropriate learning model, but, due to too little data, they may become overtrained. An extension of the problem of uncertainty estimation using BCNNs and experimental results can be found, among others, in [18,19,20].

An important issue affecting the wider use of machine learning models, particularly in healthcare, is interpretability [21]. A model is interpretable when the decisions it makes can be fully understood [22]. Unfortunately, for ordinary users, machine learning models, especially deep models, are similar to black boxes. Therefore, aiming for a wider application of artificial intelligence solutions in healthcare, a better understanding of the models’ mechanisms becomes crucial. Consequently, attempts are being made to improve the interpretability and transparency of machine learning models [23,24,25]. This is due to the need to establish a trust relationship between users and decision-making models in practical implementation applications.

The key problem of machine learning comes down to a trade-off between optimisation and generalisation. Optimisation involves tuning the model to obtain the best possible performance for the learning data. Generalisation, on the other hand, determines how well the model performs when processing new data. After a certain number of iterations (epochs) of the learning algorithm, generalisation for validation data reaches a constant level and then (mostly) starts to deteriorate. This means that over-fitting of the model to the learning data is taking place (overtraining). Various methods are used to combat over-fitting, which are generally referred to as regularisation methods [26]. Popular regularisation methods applied to learning deep convolutional neural networks include: transfer learning, dropout, data augmentation, early stopping, weights regularisation (L1, L2, max-norm), and others [27,28,29,30]. A summary and comparison of these and other regularisation methods is included in [31]. The problem of regularisation is still an area of active research, so new efficient algorithms are constantly being proposed, e.g., the two-stage training method [31].

The effects of the conducted research allow the detection of the porosity of L1 spongy tissue by analysing and classifying the spinal CT images using deep convolutional neural networks (DCNN). This is a new approach in the research on the diagnosis of osteoporosis. To the best of the authors’ knowledge, no research in which deep learning algorithms would be used to solve this problem is available. The proposed method enables to significantly simplify the stage of image pre-processing and analysis before using images to build the classifier model. The convolutional neural network is able to reveal the internal features of individual observations based on the raw data obtained from images, while ensuring high classification efficiency.

In this article, Section 2.1 describes the material used in the research and the method of selecting images. The following Section 2.2 describe the construction of the classification models, as well as the characteristics of the architecture of the network models used. Section 3 presents the obtained results of the network operation. The discussion and comparison of the research results with other works are presented in Section 4. The final part of the article summarizes the achievements and presents a plan for further work.

## 2. Materials and Methods

### 2.1. Material

The study used computed tomography images of the spine in the lumbosacral (L-S) section from 100 patients. The imaging tests used in the study were performed in the tomography laboratory located in the Independent Clinical Hospital No. 4 in Lublin (Poland). Patients were referred for examinations of the lumbosacral (L-S) spine from the orthopaedic clinic and the Hospital Emergency Department. Each patient was tested on a GE 32-row CT scan in a standard spine examination protocol, including the lumbar and sacral (L-S) vertebrae. Fifty of the patients belonged to the control group of healthy people, unaffected by osteoporosis or osteopenia. There was an equal number of patients in the group diagnosed with osteoporosis. The control group included 26 women and 24 men. The age range of the patients ranged from 53 to 77 years. The group with diagnosed osteoporosis included 33 women and 17 men aged 44 to 95 years. The patients were classified into both groups based on the description of the examination prepared by the radiologist and the measurement of the radiological density of the spongy tissue of the first lumbar spine (L1). On the basis of the literature [32], the tissue density limit was set at 120 Hounsfield units (HU).

**Figure 1:**
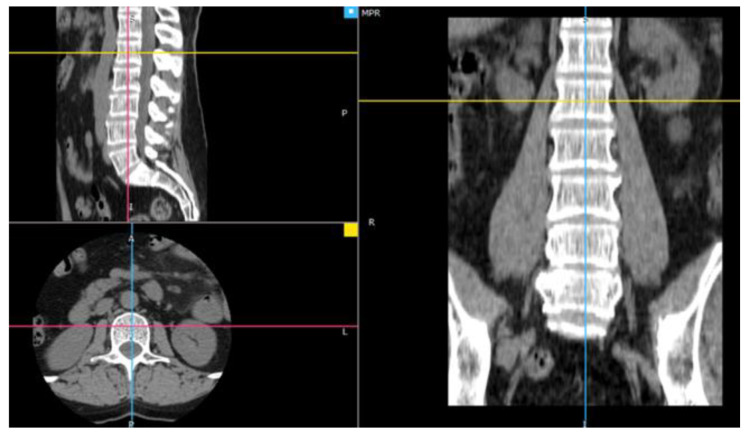
The arrangement of the axis in the centre of one of the vertebrae (image in three projections).

The source data contained images in RGB mode with a resolution of 512 × 512 pixels and was saved in the DICOM format. Soft tissue reconstruction images were used for the research. The research showed that the images derived from soft tissue reconstruction allow obtaining more accurate values of texture parameters, increasing the accuracy of classification, and offering better possibilities for diagnosing osteoporosis [33]. From the series of images, the sample sections showing the inside of the circle with the spongy essence were selected (Figure 1).

**Figure 2:**
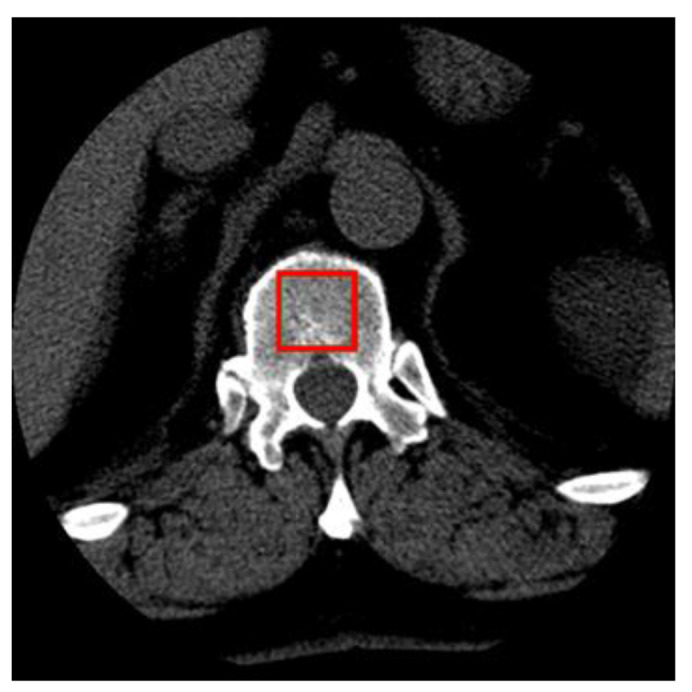
Manual selection of the spongy matter region.

Four L1 transverse images were selected from a series of studies of each patient. These images presented the sections of the vertebra closest to the middle value of its height, so that the largest possible area of the spongy tissue was visible (Figure 2). One image sample of the examined tissue was obtained from each of the selected cross-sections.

**Figure 3:**
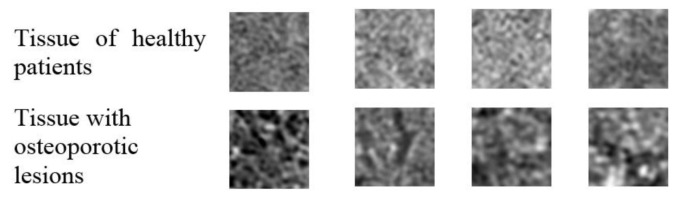
Image samples of healthy patients’ tissue and tissues with osteoporotic lesions in their original size.

The size of the extracted samples was selected to use the textured surface, potentially containing the information in the image of the transverse vertebra section, to the maximum extent. As a result, 400 samples with dimensions of 50 × 50 pixels were obtained. The sample images of the tissue from healthy and osteoporotic patients are presented below (Figure 3).

The image histogram normalisation process is one of the image preprocessing techniques frequently used for classification. As part of the studies previously carried out by the authors, it was shown that this operation reduces the classification accuracy from 4% for the TPR coefficient to 14% for ACC [34].

### 2.2. Construction of the Classification Models

The full dataset contained 400 observations, 200 of which were healthy subjects and 200 were patients diagnosed with osteoporosis. Following the procedure described in Section 2.1, both image categories were assigned labels, HEALTHY and OSTEOPOROTIC, respectively. Then, the full dataset was randomly divided into training, validation, and test subsets, with the number of observations belonging to individual classes in each of these subsets being the same. As a result of the division, the training set constituted 50% of the full set (100 observations for each class), 25% of the validation set (50 observations), and 25% of the test set (50 observations). The use of deep neural networks in research was a certain challenge, because, in the context of teaching such networks, the available number of observations was very small. Therefore, classifier models that had been previously trained on large datasets were employed. This approach is often used in the situations similar to the one considered [35]. If the dataset used to train the model was sufficiently large and general, the spatial hierarchy of the learned features can effectively act as an overall model for image processing. Such features can be helpful in solving new image processing problems, even when the problems relate to the recognition of classes other than the original classes used to train the model.

A computer with Windows 10 64-bit system, Intel Core i5-3470 3.20 GHz processor and 32 GB RAM was used to build the models. The TensorFlow 1.13.1 platform (TensorFlow, 2020), the Keras 2.2.4 [36] library, and the Python 3.7.3 programming language were employed. The calculations were performed using the GPU (NVIDIA GeForce GTX 1060 3 GB), the CUDA 9.0 platform, and the cuDNN 7.0 library. The Keras library contains many models for image processing, including Xception [37], VGG16, VGG19 [38], ResNet [39], ResNetV2 [40], InceptionV3 [41], InceptionResNetV2 [42], MobileNet [43], MobileNetV2 [44], DenseNet [35], and NASNet [45]. All these models were trained on the ImageNet collection containing approximately 1.4 million images divided into 1000 classes [46]. In the study, 6 models characterised by different topological depth were used (Table 1).

**Figure 4:**
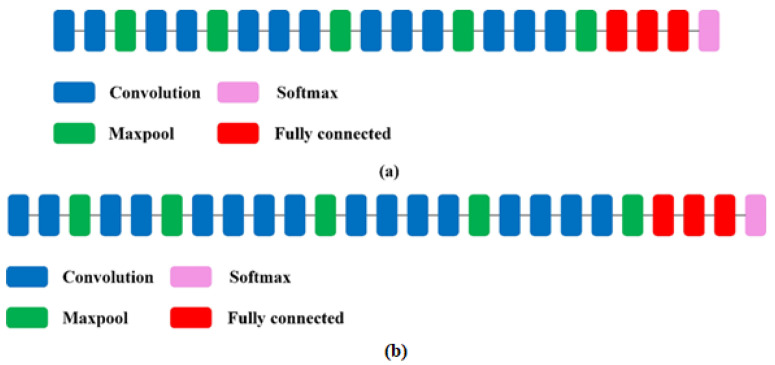
Structure of the VGG16 (**a**) and VGG19 (**b**) network models [47].

The VGG network is characterised by the smallest topological depth among the models implemented in the Keras package and a small convolutional filter of 3 × 3 pixels [38]. The VGG16 network consists of thirteen convolutional layers and three fully connected layers. The VGG19 model, on the other hand, consists of 16 convolutionary layers and three fully interconnected layers (Figure 4). Both networks use 3 × 3 pixel convolution filters. The results of the VGG network showed that a relatively small number of its layers allows for a high classification accuracy [47].

**Figure 5:**
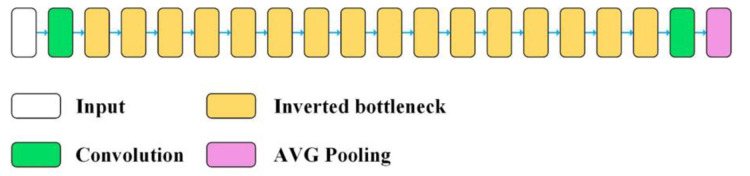
Structure of the MobileNetV2 network model [47].

MobileNetV2 is a neural network architecture that uses efficient convolutional operations called Depthwise Separable Convolution (Figure 5). Depthwise separable convolution layers spatially convolve each input channel independently and then perform a point convolution of mixing the channels (1 × 1 convolution). This is equivalent to separating learning spatial features from learning the features of individual channels. The advantages of this technique are especially important when training small models on a limited dataset [48].

**Figure 6:**
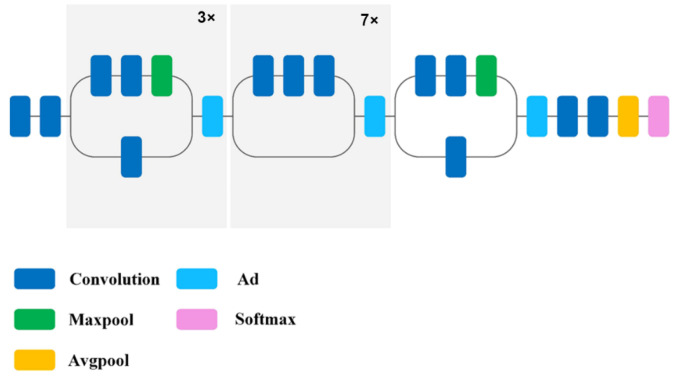
Structure of the Xception network model [47].

In the Xception network, starting blocks are based on deep, separate convolutional layers. The architecture of the Xception model is built around a linear stack of 36 deep, separate convolutional layers with linear residual connections (Figure 6). In this configuration, there are two important convolutional layers: a deep convolution layer where spatial convolution is performed independently on each input channel, and a point convolution layer where the 1 × 1 layer maps output channels to a new channel space using deep convolutions [37].

**Figure 7:**
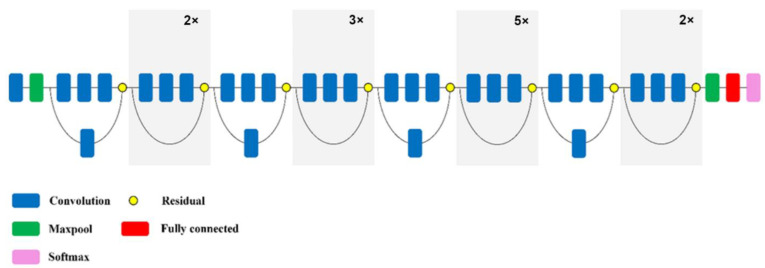
Simplified structure of the ResNet network model [47].

The ResNet model is characterised by a very deep network with 152 layers. ResNet deep configuration solves the problem of vanishing gradient by taking advantage of deep residual learning through additive identity transformations. In particular, the residual module uses a direct path between input and output, and each stacked layer matches the residual mapping rather than directly matching the desired base mapping. The optimization process is much easier for the residual mapping compared to the original unreferenced map. As with the VGG models, 3 × 3 pixel filters are most commonly used in this network. However, ResNet has fewer filters of lower complexity. The 1 × 1 convolution layers deepen the lattice and increase non-linearity by applying the ReLU function after each 1 × 1 convolution layer. In this network, fully interconnected layers are replaced by a pooling average layer. This significantly reduces the number of parameters as fully interconnected layers contain a large number of them. The network is, therefore, able to learn deeper representations of functions with fewer parameters [49]. The structure of the ResNet network is shown in Figure 7.

**Figure 8:**
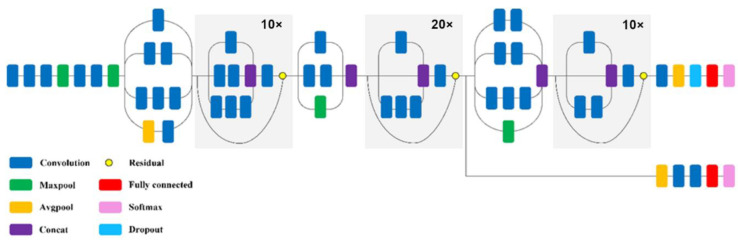
Simplified structure of the InceptionResNetV2 network model [47].

The InceptionResNetV2 network is built by integrating two models of deep convolutional networks, such as ResNet and Inception. In these networks, normalization is only applied at the top of traditional layers. The remaining modules allow you to increase the number of Inception blocks and, thus, increase the depth of the network. The most problematic issue with very deep networks is the learning phase, which can be solved with residual connections. The network rescales these connections, which is taken as an effective approach to solving the learning problem when a large number of filters (over 1000) are used in the network. In particular, the remaining variants experience instability, and the network cannot be trained when the number of filters exceeds 1000. Therefore, the residual connection scaling contributes to stabilizing the network training [50]. Figure 8 shows the compressed structure of the InceptionResNetV2 network.

**Figure 9:**
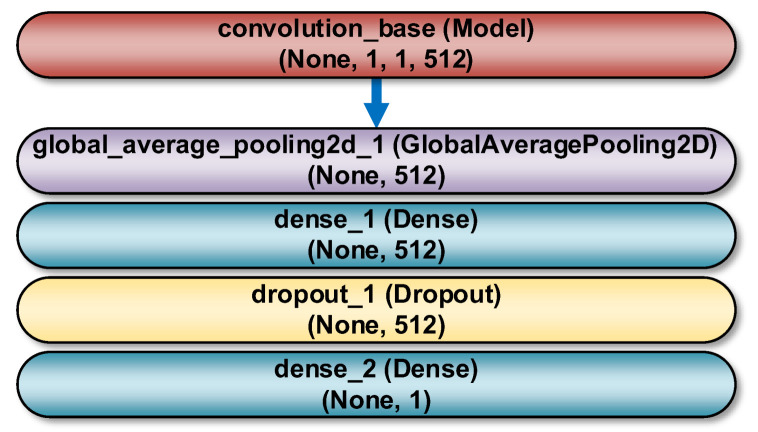
A general scheme for the implementation of a deep network that consists of a convolution base and own classifier. The meaning of the Keras classes is as follows: GlobalAveragePooling2D—a layer responsible for the global average pooling operation for spatial data; Dense—a densely connected layer; Dropout—a layer that limits the excessive adjustment of the model to the training data. The feature map has a shape (samples, height, width, and channels). The first dimension equal to None means any number of samples.

The models consisted of two parts. The first was convolutional bases, and the second was densely connected classifiers, located at the end of the network. Convolution bases were used because the representations they learned presented general concepts that are suitable for solving various image processing problems. Instead of the original classifiers, the authors used their own classifiers, made of two Dense layers. The representations they learned were specific to the set of classes tested (HEALTHY and OSTEOPOROTIC categories) on which these models were trained. Figure 9 shows the general diagram of a deep network implementation which consists of a convolutional base and a new binary classifier (2 Dense layers) added at its end.

The models were built as sequential Keras models. After convolution base, the first layer of the own classifier was GlobalAveragePooling2D. It performed pooling operations for spatial data based on a global average. Two densely connected layers were added. The first Dense layer, with 512 neurons in the output and the ReLU (Rectified Linear Unit) activation function, interpreted the features extracted by the convolution base. The second Dense layer, with 1 output neuron and the sigmoid activation function, was an output layer that predicted that an observation belonged to a specific class. One Dropout layer with the parameter rate = 0.3 was added as well, to limit the excessive adjustment of the model to the training data.

Model training was performed in two phases:

1. Feature extraction—the convolution base has been frozen, and the added dense layers, creating a new classifier, were initiated randomly and trained over a period of 200 epochs using data augmentation. Layer freezing consists of preventing the update of their weights in the training process, so that the representations previously learned by the convolution base have not been modified during training.
2. Fine tuning—the upper layers of the convolution base have been unfrozen and trained for a period of 300 epochs together with the new layers, also using data augmentation. At the end, the entire convolution base has been unfrozen.

Augmentation consisted of performing random, vertical and horizontal transformations of the image, rotation, cropping, zooming in, and reflecting half of the image in a horizontal plane. During models training, binary cross entropy as a loss function was used. The optimisation algorithm was root mean square propagation (RMSprop) with a low value of the learning parameter. It was 2 × 10^−5^ for the feature extraction phase and 10^−5^ for fine tuning. The low value of this parameter resulted from the fact that modifications of the representation of the tuned layers of the convolution base had to be minimised. Excessive changes in these values could negatively impact data representations. Accuracy was used as a measure of the training process. In the last layer of the classifier, the sigmoid activation function was used. More detailed information on the settings used during the model building process is provided in Table 2.

**Figure 10:**
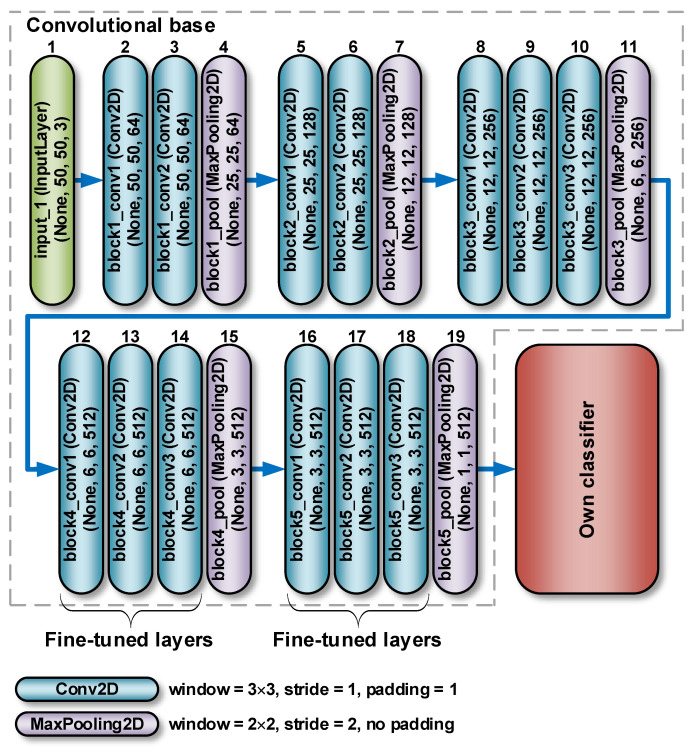
Structure of the VGG16 network.

As an implementation example, Figure 10 shows the detailed structure of the VGG16 network used. RGB images of 50 × 50 pixels are passed to the input of the network. As part of the preprocessing, a rescaling of RGB pixel intensities to the [0, 1] range was performed. The convolution base contains 13 Keras objects of the Conv2D class that form the convolution kernels. At first, the image passes through the first block built of 2 convolution layers, with ReLU activation functions. In these layers, a reception field of 3 × 3 pixels is used, the convolution stride is 1 pixel, and the padding is also equal to 1 pixel. Each layer of the first block contains 64 filters. The configuration used ensures that the spatial resolution is maintained, i.e., the size of the output activation map is the same as the dimensions of the input image. Subsequently, the activation maps are sent through a max pooling layer with a window size of 2 × 2 pixels and a stride of 2 pixels. As a result, the size of the activation maps is halved. Thus, the size of the activation maps in the output of the first block is 25 × 25 × 64. In an identical manner activation maps are sent through the second block. The only difference is that it contains 128 filters. Therefore, the size of the activation maps in the output of the second block is 12 × 12 × 128. Then, there is a third block, containing 3 convolution layers and a max pool layer. In this case, the number of filters is 256, so that, the size of the activation maps in the output of the block is 6 × 6 × 256. Further there are two more blocks (the fourth and fifth) containing 3 convolution layers each with 512 filters. The size of the activation maps in the output of the last block is 1 × 1 × 512. The convolution base is followed by own classifier (Figure 9). At its beginning, there is a layer responsible for the global average pooling operation. Next, there is a fully connected layer with the ReLU activation function, containing 512 neurons. Behind it there is a dropout layer with a rate parameter equal to 0.3, which limits the over-fitting of the model to the training data. Finally, there is a second fully connected layer, working as an output layer. It contains 1 neuron and a sigmoidal activation function.

## 3. Results

**Figure 11:**
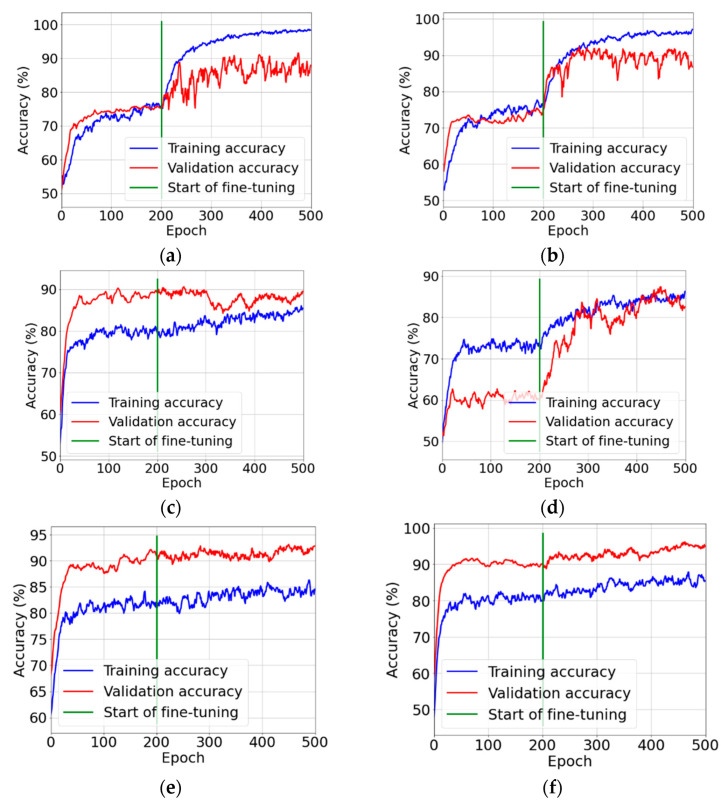
Plots of the training and validation accuracy during the stages of feature extraction and fine tuning: (**a**) VGG16; (**b**) VGG19; (**c**) MobileNetV2; (**d**) Xception; (**e**) ResNet50; and (**f**) InceptionResNetV2.

Network training was performed in two phases. They are described in more detail in Section 2.2. In the first phase (feature extraction), new network layers (a new classifier) were trained, while the entire convolution base was frozen. In the second phase (fine tuning), a certain number of final layers of the convolution base was unfrozen and trained together with the new classifier. The plots of the training and validation accuracy are shown in Figure 11.

**Figure 12:**
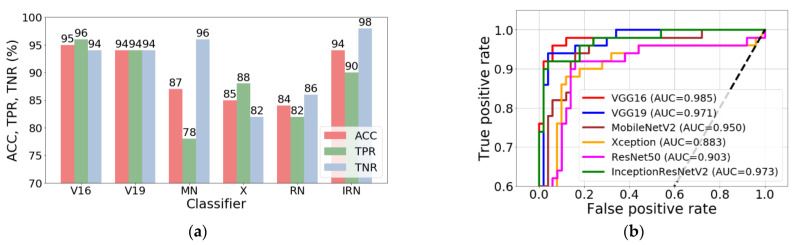
Classification results (**a**) and ROC curves and AUCs for the test set (**b**). Symbols used: V16—VGG16, V19—VGG19, MN—MobileNetV2, X—Xception, RN—ResNet50, and IRN—InceptionResNetV2.

**Figure 13:**
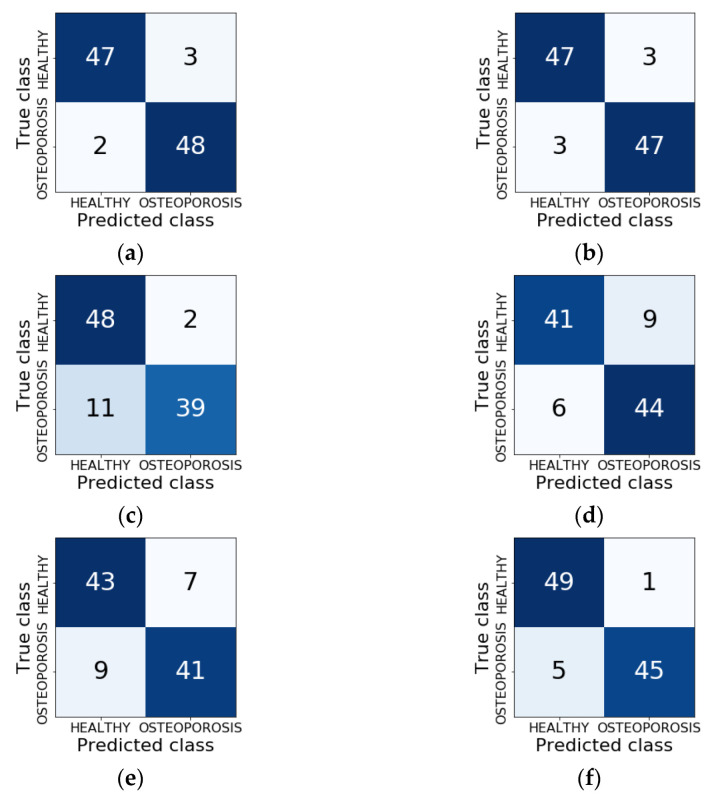
Confusion matrices: (**a**) VGG16; (**b**) VGG19; (**c**) MobileNetV2; (**d**) Xception; (**e**) ResNet50; and (**f**) InceptionResNetV2.

The final effect of the whole training process was the Keras model containing the classifier architecture, which was saved to disk as a single HDF5 file. Six such models were built during the research, one for each type of network architecture. These models were used to classify new images that did not participate in the training and validation process. Classification accuracy (ACC), sensitivity (TPR), specificity (TNR), and area under the ROC curve (AUC) were calculated (Figure 12). In addition, confusion matrices that show the distribution of correct and incorrect classification cases were built (Figure 13).

**Figure 14:**
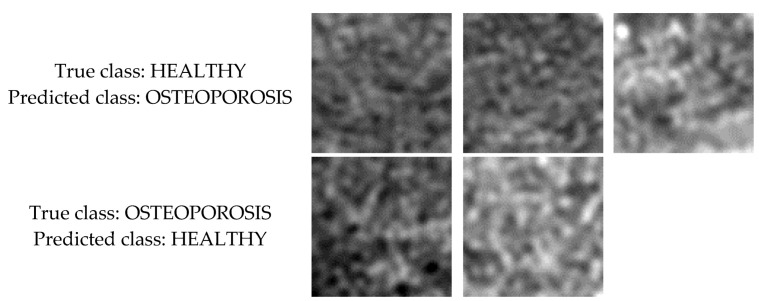
Images that were misclassified by the best VGG16 model.

As shown in Figure 3, the tissue image texture of healthy patients is smooth and homogeneous. In the case of sick patients, the texture of the tissue image is characterised by porosity and a significant number of dark spots corresponding to osteoporotic lesions. These texture properties are reflected in the feature maps built by the subsequent convolutional network layers. For the cases shown in Figure 14, the images of healthy patients (top row) contain a number of dark spots characteristic of osteoporosis. In contrast, the images of patients diagnosed with osteoporosis (bottom row) are relatively smooth and homogeneous, with a small number of darker spots. This situation makes these images more difficult cases to classify than the other images in the test set. However, this is not an abnormal situation, as when classifying biomedical images, the possibility of cases that are more difficult to recognise than the others should always be taken into account.

Table 3 shows the GPU and CPU inference time of the individual models for a single observation. A computer with Windows 10 64-bit system, Intel Core i7-4770 3.40 GHz processor, 32 GB RAM, and an NVIDIA GeForce GTX 1660 Ti 6 GB graphics card was used to measure the inference times. As expected, the fastest model was VGG16, which is due to its smallest topological depth (Table 1). Additionally, noteworthy is the ResNet50 model, which, with a relatively low inference time for the GPU (22 ms), experienced only one and a half times the inference time degradation for the CPU.

## 4. Discussion

The stage of building classifiers brought very good results. Out of the six models built, as many as three achieved the classification accuracy at the level exceeding 90% (Figure 11a). For reference, the VGG16 model achieved ACC = 95%, TPR = 96%, and TNR = 94%. At the same time, this model can be considered the best because it has the highest overall classification accuracy (95%), as well as the highest sensitivity (TPR = 96%), which is medically important for patients. The other two models are also characterised by very good quality indicators. For the VGG19 classifier, ACC = 94%, with TPR = TNR = 94%, and for the InceptionResNetV2 model ACC = 94%, TPR = 90%, and TNR = 8%. The high efficiency of the VGG16, VGG19, and InceptionResNetV2 models is also reflected in the ROC curve diagram. The AUC parameter, calculated on the basis of this plot, is equal to 0.985, 0.971, and 0.973 for the above models, respectively.

The vast majority of all quality indicators of the above-mentioned models exceeded the level of 90%. This is a very good result, taking into account the extremely small number of observations used to train the models. Each class had 200 observations, of which 100 belonged to the training set, 50 to the validation set, and 50 to the test set. For deep neural networks, these are very small datasets, because typically sets of thousands of observations are used for training them. It was possible to obtain very good results owing to the approach of using classifiers that were pre-trained on a very large ImageNet dataset. The operation of fine-tuning the top layers of the convolutional basis, together with two layers of a custom classifier added at the end of the model, proved very successful. The research confirmed that this approach may be recommended in the case of small datasets.

It should also be emphasised that the best results were obtained for the models with the smallest topological depth. The convolution base of the VGG16 model consisted of 19 layers, and in the case of the VGG19 model, there were 22 layers. Thus, the models with the lowest complexity turned out to be the most effective for a very small size of the training set. This may suggest that the number of observations was too small for the MobileNetV2, Xception, and ResNet50 models. Nevertheless, although the InceptionResNetV2 model had the greatest complexity (780 layers), it achieved very good quality indicators (ACC = 94%, TPR = 90%, TNR = 98%). A certain drawback of the method discussed is that the extraction of areas of interest was performed manually. However, this issue requires conducting separate research and will, therefore, become an area of interest in the course of further work.

A list of the results enabling to make such a comparison is provided in Table 4. As it can be seen, the results obtained are at a level comparable with the results of other authors, achieved in solving similar classification problems.

The results presented in this article are easiest to compare with the results of the studies presented in [51,52], as they concerned the same type of images, i.e., those obtained as a result of computed tomography. These studies also share the same anatomical object of interest, i.e., the spine, which is one of the elements of the human skeleton most vulnerable to osteoporosis. Due to its key role in the structure of the human body and complications after vertebral fractures leading to immobilisation of the patient and sometimes even death, the spine is the subject of many experiments aimed at preventing the final stage of the disease. As a result of the research, the outcome of which are shown in Table 4, the sensitivity of classifiers ranging from 78.83% to 98.56% obtained in study [16] with the use of the AlexNET model was obtained. In work [51], the values of the sensitivity and specificity of the used classifier were also given, which were TPR = 83.9% and TNR = 93.8%.

In contrast, study [53] presents a slightly different approach to the diagnosis of osteoporosis, but is also based on artificial neural networks. Instead of images, the research material consisted of data on the age, weight, height, and T-index of the femoral neck. The parameters shown were to be used as input data for the osteoporosis risk prediction algorithm. The achieved results were: ACC = 78.83%, AUC = 0.829, TNR = 90.12%, and TPR = 51.0%. The low value of the classifier’s sensitivity may indicate the correctness of the theory about the key importance of the analysis of bone tissue microarchitecture.

**Table 4:** Comparison with other authors.

Item No.	Role	Research Material	The Method Used	Classifier Quality Assessment Parameter
Our research	Application of deep convolutional neural networks in the diagnosis of osteoporosis	CT images of L1 spongy tissue from 100 patients (50 healthy and 50 diagnosed with osteoporosis)	VGG16, VGG19, MobileNetV2, Xception, ResNet50, InceptionResNetV2	ACC = 95%, TPR = 96%, TNR = 94%
[54]	Classification of osteoporosis on the basis of CR images of phalanges using DCNN	101 computed radiography images of phalanges	An undefined convolutional network model from the Caffe package	TPR = 64.7%, FPR = 6.51%
[51]	Identification of vertebral compression fractures caused by osteoporosis	3701 CT tests, 2681 (72%) were negative for the presence of VCF and 1020 (28%) were marked as positive for VCF,	Convolutional network and a classifier based on a recursive network	ACC = 89.1%, TPR = 83.9%, TNR = 93.8 %
[52]	Automatic detection of osteoporotic vertebral fractures on CT scans	1432 CT images of the spine	(1) a function extraction module based on CNN ResNet34 and (2) an RNN module for aggregating the extracted features and making the final diagnosis.	ACC = 89.2%, F1 score = 90.8%
[55]	Metacarpal screening for osteoporosis	4000 radiographs of the metacarpus	AlexNet	TPR = 82.4%, TNR = 95.7%
[53]	Diagnostic examination and prediction of the risk of osteoporosis in women	Age, weight, height, and T-score of the femoral neck of 1559 women	Radial basic function of artificial neural networks with the 2-4-1 architecture	ACC = 78.83%, AUC = 0.829, TPR = 51.0%, TNR = 90.12%
[56]	Prediction of osteoporosis from simple hip radiography	1012 simple hip radiographs	VGG16 model	ACC = 81.2%, TPR = 91.1%, TNR = 68.9%, PPV = 78.5%, NPV = 86.1%
[16]	Predicting osteoporosis based on the mandibular cortical index on panoramic radiographs	Panoramic radiographs of mandibular 744 female patients	AlexNET, GoogleNET, ResNET-50, SqueezeNET, and ShuffleNET deep-learning models	ACC = 81.14%(AlexNET), ACC = 88.94% (GoogleNET), ACC = 98.56% (AlexNET), ACC = 92.79% (GoogleNET)

**Figure 15:**
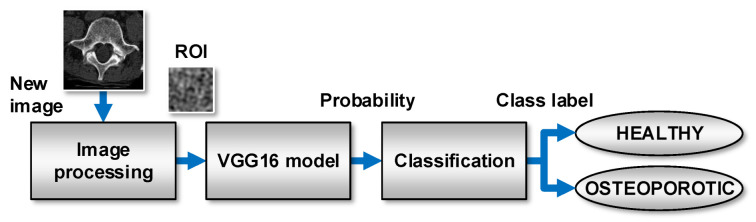
Process flow during the prediction of a class of new images.

**Figure 16:**
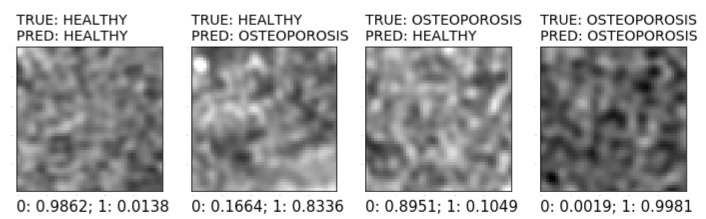
Various prediction results of sample images.

Figure 15 shows the general scheme of the system for predicting new images using the VGG16 model, which achieved the highest quality indicators (ACC = 95%, TPR = 96%, and TNR = 94%). At the same time, this model is characterised by the lowest topological complexity (the convolution base contains 19 layers). After ROI extraction, new observations are fed to the input of the VGG16 model. The model analyses the data and calculates the probability of belonging to a positive class (OSTEOPOROTIC). If this probability is equal to or greater than 0.5, the classification module assigns the OSTEOPOROTIC label to the observations. Otherwise, the observation is labelled HEALTHY. Figure 16 shows the various possible prediction results for the sample test images. These results were obtained using a prototype system for the diagnosis of osteoporosis, which uses the VGG16 model for prediction.

## 5. Conclusions

The article presents an algorithm for the classification of computed tomography images of the spongy tissue of the lumbar spine using six different convolutional neural network models. Out of the convolutional network models built, as many as three achieved the classification accuracy at a level exceeding 90%. The VGG16 network model turned out to be the best, because it is characterised by the highest classification accuracy (ACC = 95%) and the highest sensitivity (TPR = 96%), which is very important from the medical point of view. This means that properly constructed and trained convolutional neural networks can be the basis for the creation of an effective method for the diagnosis of spinal osteoporosis, through the classification of CT images of spongy tissue.

The plan for further work includes the attempts to create a complete osteoporosis diagnostic system based on the CT images of the spine and other bone elements exposed to this disease. The practical implementation of the proposed prototype osteoporosis diagnostic system based on the VGG16 convolutional network model should be preceded by tests on a larger number of patients and creation of an algorithm for automatic segmentation of tissue image samples from the sequence of images in the DICOM format directly from the CT scanner.

## Figures and Tables

**Table 1:** Models for image classification with weights trained on ImageNet that have been used in the study (CB—Convolution Base).

No.	Model	The Number of Layers of the CB	Fine-Tuned Layers of the CB	Type of Fine-Tuned Layers
1	VGG16	19	12–19	2D convolution
2	VGG19	22	13–22	2D convolution
3	Xception	132	117–132	depth-wise separable 1D and 2D convolution, batch normalization
4	MobileNetV2	155	136–155	2D convolution, depth-wise convolution, batch normalization
5	ResNet50	175	150–175	2D convolution, batch normalization
6	InceptionResNetV2	780	631–780	2D convolution, batch normalization

**Table 2:** Settings used during the models building process.

	Parameter	Value
Training	epochs	200 (feature extraction), 300 (fine tuning)
	batch size	5
Model	loss function	binary cross entropy
	optimizer	root mean square propagation (RMSprop)
	metrics	accuracy
Optimizer	learning rate	2 × 10^−5^ (feature extraction), 10^−5^ (fine tuning)
	rho	0.9
	momentum	0.0
	epsilon	10^−7^
	centred	False
Data augmentation	rotation range	40°
	width shift range	0.2
	height shift range	0.2
	shear range	0.2
	zoom range	0.2
	horizontal flip	True

**Table 3:** Inference time for a single observation.

Model	GPU Inference Time [ms]	CPU Inference Time [ms]	Latency Increasing (*t_CPU_* / *t_GPU_*)
VGG16	5.6	20.6	3.7
VGG19	7.0	26.1	3.7
Xception	25.2	58.0	2.3
ResNet50	22.0	33.9	1.5
MobileNetV2	21.1	65.0	3.1
InceptionResNetV2	93.8	115.0	1.2

## Data Availability

The data presented in this study are available on request from the corresponding author. The data are not publicly available due to restrictions regarding privacy.
